# The Benzimidazole-Based Anthelmintic Parbendazole: A Repurposed Drug Candidate That Synergizes with Gemcitabine in Pancreatic Cancer

**DOI:** 10.3390/cancers11122042

**Published:** 2019-12-17

**Authors:** Rosalba Florio, Serena Veschi, Viviana di Giacomo, Sara Pagotto, Simone Carradori, Fabio Verginelli, Roberto Cirilli, Adriano Casulli, Antonino Grassadonia, Nicola Tinari, Amelia Cataldi, Rosa Amoroso, Alessandro Cama, Laura De Lellis

**Affiliations:** 1 Department of Pharmacy, G. d’Annunzio University of Chieti-Pescara, 66100 Chieti, Italy; rosalba.florio@unich.it (R.F.); veschi@unich.it (S.V.); viviana.digiacomo@unich.it (V.d.G.); fabio.verginelli@unich.it (F.V.); amelia.cataldi@unich.it (A.C.); rosa.amoroso@unich.it (R.A.); laura.delellis@unich.it (L.D.L.); 2 Department of Medical, Oral and Biotechnological Sciences, G. d’Annunzio University of Chieti-Pescara, 66100 Chieti, Italy; sara.pagotto@unich.it (S.P.); antonino.grassadonia@unich.it (A.G.); nicola.tinari@unich.it (N.T.); 3 Center for Advanced Studies and Technology (CAST), University “G. d’Annunzio” of Chieti-Pescara, 66100 Chieti, Italy; 4 Centro nazionale per il controllo e la valutazione dei farmaci, Istituto Superiore di Sanità, 00161 Rome, Italy; roberto.cirilli@iss.it; 5 WHO Collaborating Centre for the Epidemiology, Detection and Control of Cystic and Alveolar Echinococcosis (in Animals and Humans), Istituto Superiore di Sanità (ISS), 00161 Rome, Italy; adriano.casulli@iss.it; 6 European Union Reference Laboratory for Parasites, Istituto Superiore di Sanità (ISS), 00161 Rome, Italy

**Keywords:** drug repurposing, synergism, combined treatments, pancreatic ductal adenocarcinoma, mitotic catastrophe, benzimidazoles

## Abstract

Pancreatic cancer (PC) is one of the most lethal, chemoresistant malignancies and it is of paramount importance to find more effective therapeutic agents. Repurposing of non-anticancer drugs may expand the repertoire of effective molecules. Studies on repurposing of benzimidazole-based anthelmintics in PC and on their interaction with agents approved for PC therapy are lacking. We analyzed the effects of four Food and Drug Administration (FDA)-approved benzimidazoles on AsPC-1 and Capan-2 pancreatic cancer cell line viability. Notably, parbendazole was the most potent benzimidazole affecting PC cell viability, with half maximal inhibitory concentration (IC_50_) values in the nanomolar range. The drug markedly inhibited proliferation, clonogenicity and migration of PC cell lines through mechanisms involving alteration of microtubule organization and formation of irregular mitotic spindles. Moreover, parbendazole interfered with cell cycle progression promoting G2/M arrest, followed by the emergence of enlarged, polyploid cells. These abnormalities, suggesting a mitotic catastrophe, culminated in PC cell apoptosis, are also associated with DNA damage in PC cell lines. Remarkably, combinations of parbendazole with gemcitabine, a drug employed as first-line treatment in PC, synergistically decreased PC cell viability. In conclusion, this is the first study providing evidence that parbendazole as a single agent, or in combination with gemcitabine, is a repurposing candidate in the currently dismal PC therapy.

## 1. Introduction

Pancreatic cancer (PC) is one of the most fatal tumors worldwide, with a very poor overall survival and a 5-year survival rate of less than 6% [1]. Incidence and mortality rates for PC are rising according to GLOBOCAN 2018 estimates [2]. Its dismal prognosis is due to the small fraction of patients eligible to radical surgery (15–20%) and to the limited response to standard chemotherapy regimens [1]. Currently, chemotherapeutic options include gemcitabine monotherapy, or combination strategies based on gemcitabine plus nab-paclitaxel, or FOLFIRINOX (5-fluorouracil, leucovorin, irinotecan and oxaliplatin). However, even combination strategies offer a limited survival advantage for PC patients as compared to single agents and they are often accompanied by serious side effects. Thus, more effective and less toxic drugs to be employed in PC treatment are needed.

Natural and synthetic molecules, as well as Food and Drug Administration (FDA)-approved drugs candidate for repurposing in oncology are currently being considered as single compounds, or in combination for PC therapy [3,4,5,6,7,8]. Repurposing of approved non-anticancer drugs in cancer therapy may have several advantages, including good safety profiles and recognized pharmacokinetic properties in term of absorption, metabolism and toxicity, which may accelerate the clinical translation in cancer therapy of preclinical results obtained with these drugs [9,10,11]. Some members of benzimidazole-based anthelmintic family, such as albendazole, mebendazole and flubendazole, are being considered for repurposing in cancer therapy [12]. These drugs are widely used in animals and humans as safe and effective treatments for intestinal parasites [13,14,15], but there is also evidence of their antitumor effects in melanoma, leukemia, medulloblastoma, breast and colorectal cancers [16,17,18,19,20,21,22]. Interestingly, very little is known about the effects of these drugs in PC. Previous studies provided preliminary evidence that albendazole and mebendazole may affect PC viability [23,24], but the effects of other benzimidazoles have not been tested in this tumor thus far. Moreover, it is unknown whether benzimidazole-based anthelmintics may enhance the effects of drugs approved for PC treatment, since studies combining these agents are lacking.

In the present study, we explored the effects of FDA-approved benzimidazole-based anthelmintics fenbendazole, mebendazole, oxibendazole and parbendazole in PC cell lines. Parbendazole, whose antitumor potential has not been investigated before in PC, emerged as the most potent compound in reducing viability of two PC cell lines with distinct genetic backgrounds. Moreover, the drug markedly inhibited growth, abolished clonogenic activity, altered microtubule organization, affected migration, drastically perturbed cell cycle, promoted apoptosis and induced DNA damage response in PC cell lines. Notably, combinations of the drug with the first-line treatment gemcitabine synergistically affected PC cell viability, indicating that parbendazole, as a single agent or in combination, is a candidate for repurposing in pancreatic cancer, which may be relevant for clinical translation.

## 2. Results

### 2.1. Parbendazole Has the Lowest IC_50_ in the Panel of Tested Benzimidazoles

**Figure 1:**
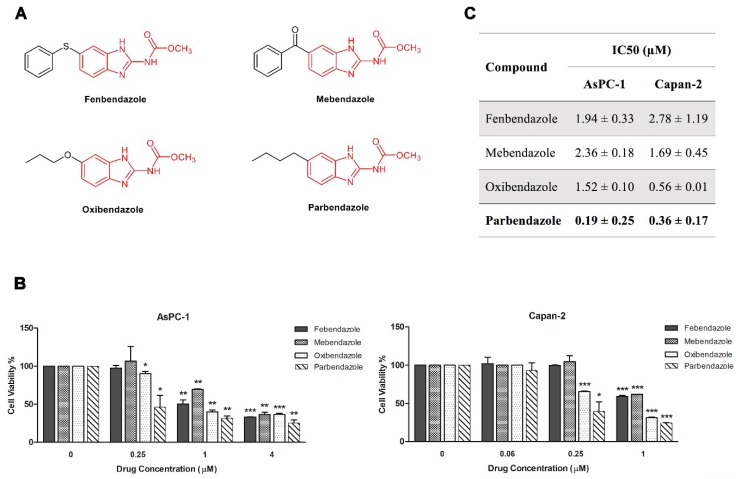
Parbendazole is more effective than fenbendazole, mebendazole and oxibendazole on PC cell viability. (**A**) Chemical structures of the four tested benzimidazole-based anthelmintics. (**B**) Pancreatic cancer cell viability was assessed by 3-(4,5-dimethyl-2-thiazolyl)-2,5-diphenyl-2*H*-tetrazolium bromide (MTT) after treatment of AsPC-1 and Capan-2 cell lines with fenbendazole, mebendazole, oxibendazole, parbendazole or with vehicle control at the indicated concentrations for 72 h. (**C**) Cell viability IC_50_ values for fenbendazole, mebendazole, oxibendazole and parbendazole. Data shown are means ± SD of two independent experiments with quintuplicate determinations. * Statistically significant differences as compared to vehicle (control) (* *p* < 0.05; ** *p* < 0.01; *** *p* < 0.001).

We analyzed the effects of four benzimidazole-based anthelmintics, namely fenbendazole, mebendazole, oxibendazole and parbendazole, on the viability of AsPC-1 and Capan-2 cell lines (Figure 1). The four drugs have different substituents at the 5^th^ position of the benzimidazole nucleus, as shown in Figure 1 (Figure 1A). All drugs significantly affected viability of both PC cell lines in a dose-dependent fashion, with half maximal inhibitory concentration (IC_50_) values (±SD) ranging from 0.19 (±0.25) µM to 2.36 (±0.18) µM in AsPC-1 and from 0.36 (±0.17) µM to 2.78 (±1.19) µM in Capan-2 (Figure 1B,C). These IC_50_ values were all in the range or even lower than the therapeutic plasma concentrations reached after administration of standard dosages [17,25,26,27,28]. Notably, parbendazole showed the lowest IC_50_ values as compared to other benzimidazoles tested in both cell lines (Figure 1C), suggesting that the specific substituent at the C5 might be relevant for anti-proliferative activities. To evaluate lipophilicity of the anthelmintic drugs, we obtained the calculated logarithm of the partition coefficient for *n*-octanol/water (CLogP) values by ChemBioDraw Ultra^®^ 12.0. The lipophilicity resulted comparable among the four compounds, ranging from 3.072 to 4.182 (CLogP values for fenbendazole 4.182, mebendazole 3.076, oxibendazole 3.072 and parbendazole 3.795) and did not appear to correlate with their activity. Considering that parbendazole was the most potent compound in inhibiting PC cell viability among the tested benzimidazoles, we selected this drug for further characterization of its antitumor potential.

### 2.2. Parbendazole Hampers Growth and Clonogenicity of PC Cell Lines

**Figure 2:**
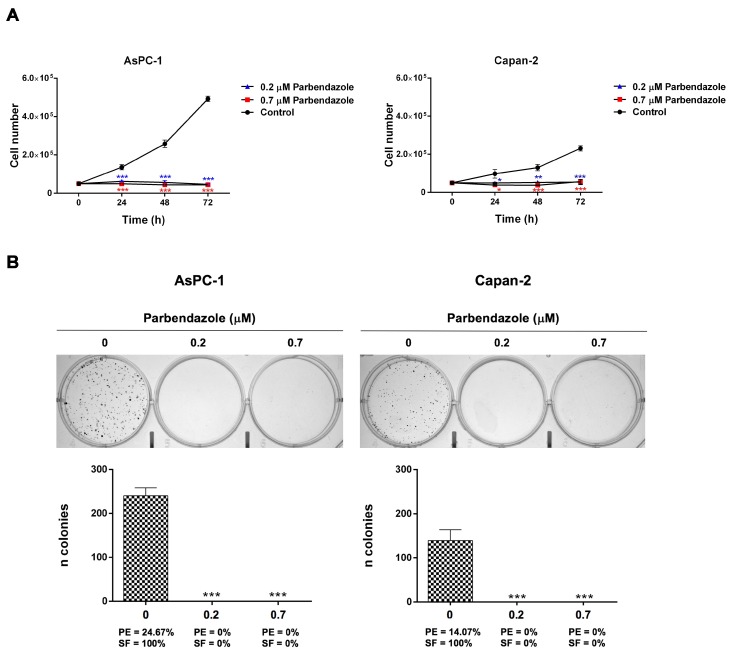
Parbendazole abolishes growth and clonogenicity of PC cell lines. (**A**) Cell growth was assessed by trypan blue exclusion test over a 72-h time course treatment with 0.2 μM and 0.7 μM parbendazole, or with vehicle control. Data shown are the means (±SD) of three independent experiments (* *p* < 0.05; ** *p* < 0.01; *** *p* < 0.001). (**B**) Representative plates of colony formation assays for AsPC-1 and Capan-2 (top). Values represented in the histograms (bottom) are the means (±SD) of three independent experiments (*** *p* < 0.001). PE: plating efficiency [(# of colonies formed/# of cells plated) × 100]; SF: surviving fraction [# of colonies formed × 100/(# of cells plated × PE of control vehicle)].

We analyzed the impact of parbendazole on AsPC-1 and Capan-2 cell growth and clonogenicity (Figure 2). Parbendazole at lower and higher concentrations drastically reduced cell growth of PC cell lines at 24, 48 and 72 h, as compared to vehicle control (Figure 2A). Clonogenicity of AsPC-1 and Capan-2 was totally abolished by parbendazole both at lower and higher concentrations, as compared to vehicle control (Figure 2B). These findings indicate that, even at the lowest concentration tested, parbendazole dramatically affects growth and clonogenic ability of pancreatic cancer cells.

### 2.3. Parbendazole Alters Mitotic Spindles Formation in PC Cells

**Figure 3:**
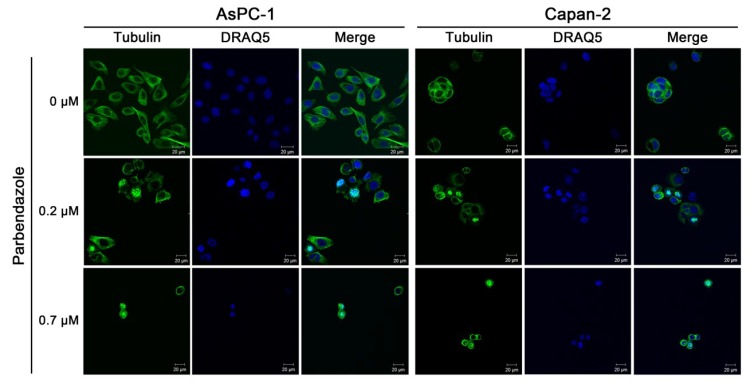
Parbendazole alters mitotic spindles formation. Immunofluorescence of PC cells (AsPC-1, left panels; Capan-2, right panels) stained using anti-α-tubulin antibody (green) and 1,5-bis{[2-(dimethylamino)ethyl]amino}-4,8-dihydroxyanthracene-9,10-dione (DRAQ5) (blue, nuclear staining). Both cell lines were treated for 24 h with 0.2 µM and 0.7 µM parbendazole, or with vehicle control. Representative pictures of two independent experiments are shown (scale bar = 20 µm).

Based on evidence suggesting that the alteration of microtubule dynamics may contribute to the antitumor potential of benzimidazoles [16,18,19,22], we investigated whether parbendazole could affect microtubule network in AsPC-1 and Capan-2 cell lines by anti-α-tubulin immunofluorescence (Figure 3). In untreated cells, microtubules were distributed in an ordered network of long filaments (Figure 3). Conversely, even with low concentrations of parbendazole, most cells lost their typical arrangement, showing a compact and round morphology with formation of aberrant spindles, instead of bipolar mitotic spindles (Figure 3). These results indicate that parbendazole alters tubulin distribution causing the formation of irregular mitotic spindles in PC cells.

### 2.4. Parbendazole Affects Cell Cycle Altering DNA Content and Size of PC Cells

**Figure 4:**
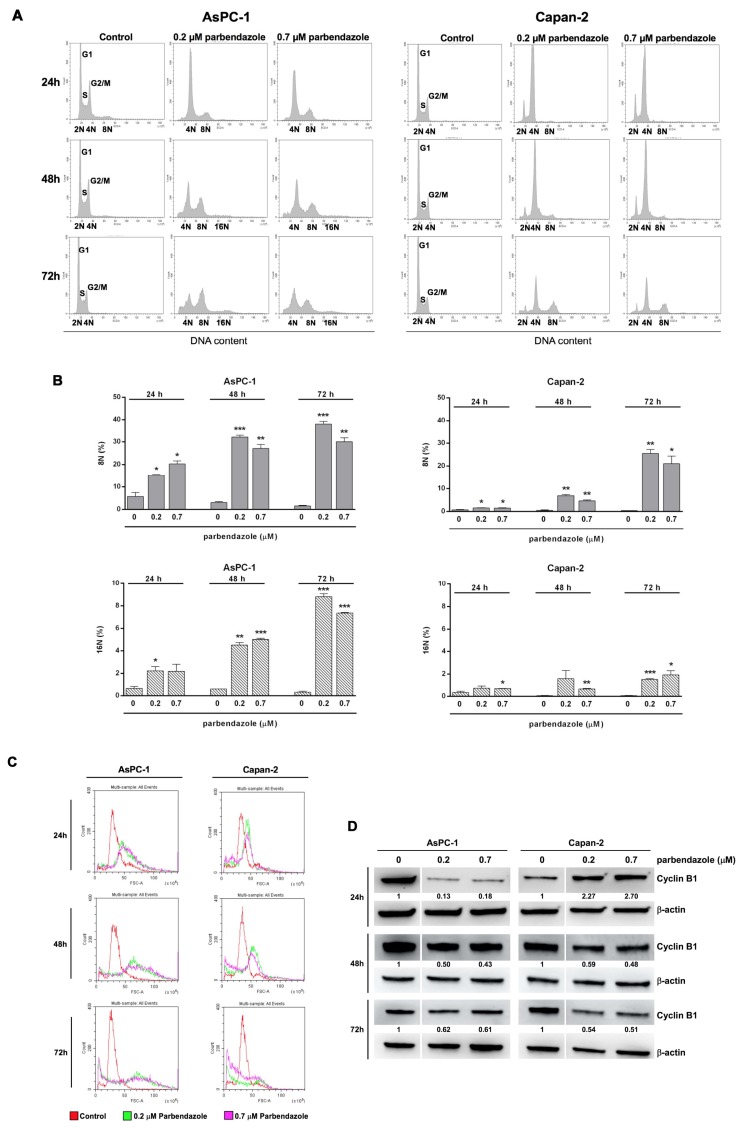
Parbendazole affects cell cycle, ploidy and size of PC cells. (**A**) Representative DNA distribution histograms of AsPC-1 and Capan-2 cell lines incubated with 0.2 μM, 0.7 μM parbendazole or with vehicle control for 24, 48 and 72 h, as measured by flow cytometry. (**B**) Histograms show the percentages of 8N and 16N cells after parbendazole treatment for 24, 48 and 72 h, as determined by flow cytometry. Data shown are the means (±SD) of two independent experiments (* *p* < 0.05; ** *p* < 0.01; *** *p* < 0.001). (**C**) Histogram plots showing cell size shifts after parbendazole treatment for 24, 48 and 72 h, as detected by flow cytometry analysis of forward scatter. (**D**) Representative western blots showing cyclin B1 expression in AsPC-1 and Capan-2 treated with 0 μM, 0.2 μM or 0.7 μM parbendazole for 24, 48 and 72 h. β-actin was included as loading control. Numbers below blots refer to the densitometric analysis of the immunoreactive bands and represent the fold change in protein expression normalized to β-actin.

Considering that tubulin is essential in cell division and that disorganized microtubule formation prevents cell cycle progression [18,21,29], we analyzed the effects of parbendazole on the PC cell cycle. Flow cytometry analysis indicated that parbendazole induced a profound perturbation of the cell cycle in both PC cell lines (Figure 4). A large proportion of AsPC-1 cells underwent cell cycle arrest in the G2/M phase, with a sharp increase in 4N cells after treatment with both concentrations of parbendazole (0.2 μM or 0.7 μM) for 24 h. This arrest was accompanied both by a severe decrease in the proportion of 2N cells in G1 phase and by the emergence of octaploid G2/M cells (8N) (Figure 4A,B). After 48 and 72 h of treatment, G1 phase abolishment and G2/M arrest were maintained, with a significant increase of the proportion of octaploid (8N) and even hexadecaploid (16N) cells (Figure 4A,B). Similarly, parbendazole-treated Capan-2 cells underwent cell cycle arrest in G2/M phase at 24 h, which was maintained at 48 and 72 h, but in this case a small proportion of diploid cells in G1 phase (2N) was preserved at all time points (Figure 4A,B). In addition, parbendazole induced an increasing population of octaploid (8N) cells at 24 h through 72 h, whereas the population of 16N cells was smaller, nevertheless significantly increased, at 72 h (Figure 4A,B). Notably, in both cell lines the increase in the proportion of polyploid cells (8N,16N) induced by parbendazole treatment was paralleled by a size shift, as indicated by flow cytometry analysis of forward scatter, with the appearance of enlarged cells, more evident in AsPC-1 at 24 and 48 h (Figure 4C).

Western blot analysis showed that cell cycle perturbation induced by parbendazole was associated with marked alterations in cyclin B1 expression, a protein involved in the control of G2/M checkpoint (Figure 4D and Figure S1). In accordance with the different dynamics of cell cycle perturbation revealed by flow cytometry in the two cell lines, distinct patterns of cyclin B1 expression were observed. In AsPC-1, parbendazole induced a decrease in cyclin B1 expression at all time points (Figure 4D and Figure S1). This result was in line with the increasing accumulation of polyploid cells at the corresponding time points indicated by flow cytometry. In Capan-2, parbendazole induced a transient accumulation of cyclin B1 at 24 h, followed by a decrease in its expression at later time points (Figure 4D and Figure S1). This dynamics of cyclin B1 expression was in line with flow cytometry analysis that revealed a prolonged G2/M arrest in Capan-2, followed by a gradual increase in the proportion of polyploid cells at later time points. A similar early transient accumulation of cyclin B1, followed by its decreased expression was previously described in lung carcinoma cells undergoing G2/M arrest after fenbendazole treatment [26]. Overall, in both AsPC-1 and Capan-2 parbendazole induced cell cycle arrest, appearance of enlarged polyploid cells and altered cyclin B1 expression. The slight differences in the dynamics of these events between the two PC cell lines is likely correlated to inherent differences in genetic profiles.

### 2.5. Parbendazole Promotes Apoptosis and DNA Damage in PC Cells

**Figure 5:**
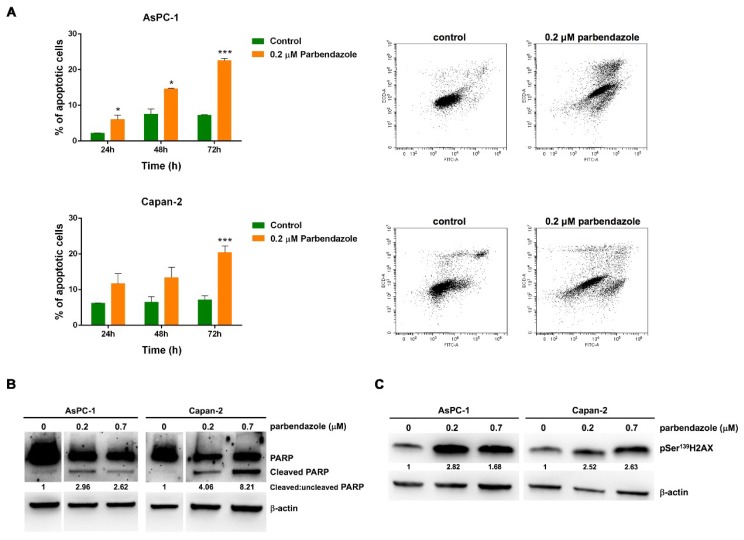
Parbendazole induces apoptosis and DNA damage in PC cell lines. (**A**) Cells were treated with parbendazole, or with vehicle control for 24, 48 and 72 h. Values represented in the histograms (left) are the means (±SD) of at least three independent flow cytometry experiments (* *p* < 0.05; *** *p* < 0.001). Representative dot plots (right) of flow cytometry experiments after a 72-h treatment with parbendazole, or with vehicle control. (**B**) Representative western blots showing PARP and cleaved PARP protein expression in AsPC-1 and Capan-2 treated with 0 μM, 0.2 μM or 0.7 μM parbendazole. Ratios of cleaved: uncleaved PARP are indicated. (**C**) Representative western blots showing levels of H2AX phosphorylation at Ser^139^ in AsPC-1 and Capan-2 treated with 0 μM, 0.2 μM or 0.7 μM parbendazole. β-actin was included as loading control. Numbers below blots refer to the densitometric analysis of the immunoreactive bands and represent the fold change in protein expression normalized to β-actin.

To evaluate whether parbendazole-induced decrease in PC cell viability and proliferation could associate with apoptosis induction, we analyzed Annexin-V staining using flow cytometry (Figure 5). In AsPC-1, parbendazole induced significant apoptosis at 24 and 48 h, with a more marked increase of apoptotic cells at 72 h as compared to vehicle control (Figure 5A). In Capan-2, a significant induction of apoptosis was detected after parbendazole treatment for 72 h, as compared to vehicle control (Figure 5A). In line with the results obtained by flow cytometry, western blot analysis showed that parbendazole treatment induced a marked increase of poly-(ADP-ribose) polymerase (PARP) cleavage in the two PC cell lines, as compared to vehicle control (Figure 5B and Figure S2). Overall, flow cytometry and western blot analyses consistently indicated that parbendazole promoted apoptosis in PC cell lines.

Considering that DNA damage could relate with apoptosis and that in a previous study parbendazole was shown to induce such damage in osteosarcoma and cervix adenocarcinoma cell lines [30], we explored whether parbendazole treatment could elicit DNA damage response in AsPC-1 and Capan-2 cell lines (Figure 5C and Figure S3). Immunoblot analysis of pSer^139^H2AX showed that parbendazole induced a sharp activation of this sensitive DNA damage marker.

Taken together, these findings indicate that DNA damage and apoptosis contribute to the reduced viability and proliferation induced by the drug in PC cells.

### 2.6. Parbendazole Impairs PC Cell Migration

**Figure 6:**
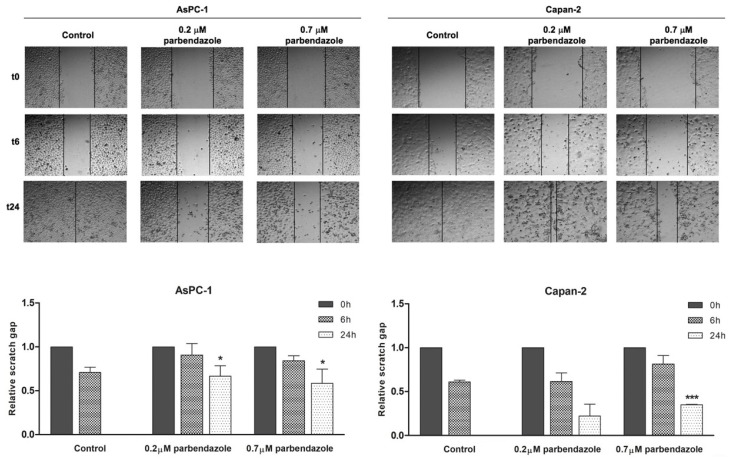
Parbendazole affects PC cell migration. Representative wound-healing assay pictures for AsPC-1 and Capan-2 treated with parbendazole, or with vehicle control (upper panels). Pictures of PC cells were taken at 0, 6 and 24 h post-scratching to analyze the dynamics of wound closure (vertical black lines indicate wound edges). Histograms (bottom panels) represent cell migration in two independent experiments expressed as relative scratch gap. * Statistically significant differences as compared to vehicle (* *p* < 0.05; *** *p* < 0.001).

Considering that microtubules have a crucial role in cell migration [31], we evaluated the effects of parbendazole on AsPC-1 and Capan-2 cancer cell migration by wound-healing assays (Figure 6). Cell migration was monitored over time after incubation in starvation medium with 0.2 μM or 0.7 μM parbendazole, or with vehicle. Parbendazole affected cell migration in both AsPC-1 and Capan-2 (Figure 6). In AsPC-1, there was a marked and significant impairment of cell migration at 24 h, with higher and lower concentrations of the drug. In Capan-2 the impairment of cell migration was less marked, but a significant reduction was observed at 24 h, with the highest concentration of parbendazole.

### 2.7. Combinations of Parbendazole and Gemcitabine Synergistically Affect PC Cell Viability

**Figure 7:**
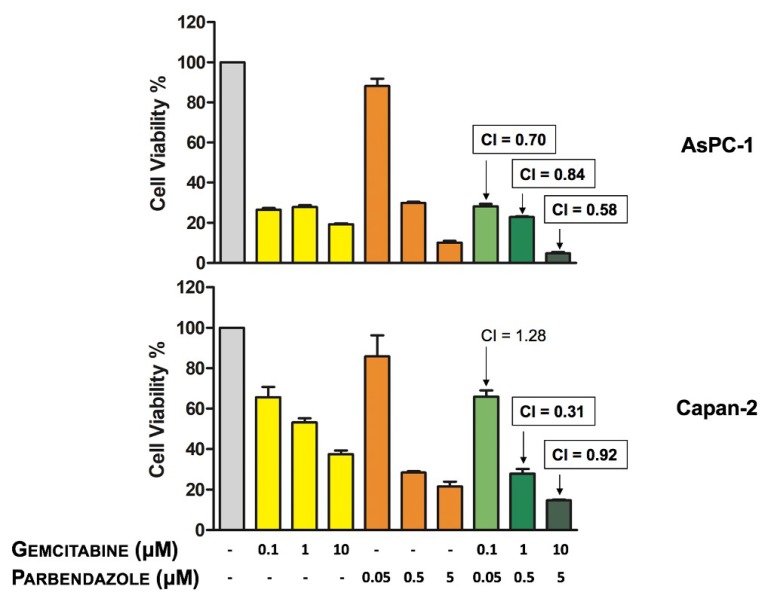
Effect of parbendazole and gemcitabine combinations on PC cell viability. Cell viability was assessed by MTT after a 72-h incubation of AsPC-1 and Capan-2 with parbendazole and gemcitabine, as single agents or in combination, at the indicated concentrations. Combination indexes (CIs) were calculated by CompuSyn software. Synergistic combinations (CI < 1) are boxed and in bold. Values represented in the histograms are the means (±SD) of two independent experiments with quintuplicate determinations.

To test whether parbendazole could be conveniently combined with gemcitabine, a drug employed as first-line therapy in PC, we analyzed the effect of the two drugs in combination on PC cell viability. Overall, combined treatments affected PC cell viability in a dose-dependent manner (Figure 7). In both cell lines, the reduction of cell viability was particularly marked using the combination with the highest concentrations of the drugs. Interactions between parbendazole and gemcitabine in the two cell lines were analyzed by CompuSyn software [32]. In AsPC-1, all combined treatments resulted synergistic, as assessed by combination indexes (CI < 1) (Figure 7). In Capan-2, CompuSyn indicated that two drug combinations had synergistic effects (CI < 1), whereas one combination appeared moderately antagonistic (CI > 1) (Figure 7). These results indicate that in both PC cell lines parbendazole and the PC-approved drug gemcitabine might be effectively and synergistically combined.

## 3. Discussion

Pancreatic cancer (PC) is one of the most lethal human cancers, but presently, both standard chemotherapy regimens and targeted agents provide unsatisfactory responses in terms of survival for patients with advanced PC [33]. This issue may be partly explained by a complex cross-talk between PC cells and microenvironment components, such as stromal and immune cells, as well as soluble proteins, including growth factors and cytokines, which play a crucial role in conferring chemoresistance, promoting tumor growth, metastatic dissemination and epithelial-mesenchymal transition (EMT), supporting immunoescape and interfering with drug delivery [34,35,36]. Thus, more effective therapeutic strategies and agents are needed to improve pancreatic cancer treatment. Development of novel anticancer compounds is often challenging and costly in time and resources. Moreover, novel drugs may fail after their translation from the bench to the bedside due to unexpected lack of efficacy or safety problems. In this regard, repurposing of FDA-approved non-anticancer drugs in oncology is considered a promising strategy and is an active field of research [9,10].

Benzimidazole-based anthelmintics are among drugs considered for repurposing in cancer treatment [12], but studies on the effects of these compounds in PC and on their interaction with agents approved in PC therapy are lacking. Here, we investigated the effects of benzimidazoles in PC cell lines. In a panel of four benzimidazoles screened, parbendazole was the most effective in reducing PC cell viability and was active in the nanomolar range. The variability of the IC_50_ values obtained with the four tested compounds is likely to be related to the distinct substituents at C5 of the benzimidazole nucleus, suggesting that these substituents play an important role in the anti-proliferative activity. In particular, linear alkylic (parbendazole) or alkyloxy (oxibendazole) chains are preferred as compared to an aryl ring connected through a sulfur (fenbendazole) or a carbonyl (mebendazole) bridge in mediating anti-proliferative effects. This implies that a higher conformational freedom may be pivotal in this portion of the molecule. Conversely, the effects of the four benzimidazoles on PC cell viability did not appear to correlate with the lipophilicity of the anthelmintic drugs. Despite the different activities of the compounds, all of these drugs affected PC viability with IC_50_ values in the range achieved by standard therapeutic dosages [17,25,26,27,28].

Parbendazole drastically reduced cell growth and abolished clonogenic capacity of PC cell lines, indicating a strong effect on PC cell proliferation and on self-renewal, even at the lowest concentration tested. In addition, parbendazole markedly altered the intracellular microtubule network in PC cells, leading to the formation of aberrant mitotic spindles. Considering that microtubules are required for separation of duplicated chromosomes during cell division, we evaluated the effect of parbendazole on PC cell cycle progression. The drug profoundly perturbed cell cycle by inducing G2/M cell cycle arrest and abnormal ploidy in AsPC-1 and Capan-2 cells. Interestingly, slight differences between the two PC cell lines were revealed by flow cytometry and western blot analyses. In AsPC-1, we observed a large population of cells undergoing mitotic slippage without cell division to become polyploid, with the appearance of a significant percentage of octaploid and hexadecaploid cells already at 24 h, which increased at later time points. This altered ploidy was accompanied by a gradual increase in cell size, as indicated by flow cytometry analysis of cell scattering. Conversely, in Capan-2 parbendazole induced a more clear-cut and prolonged cell cycle arrest in G2/M phase, accompanied by the slower emergence of a small fraction of polyploid cells representing a population of cells that exited mitotic arrest without cell division, as observed in AsPC-1. In line with the prolonged cell cycle arrest in G2/M observed by flow cytometry in Capan-2, western blot analysis showed that parbendazole induced transient accumulation of the cyclin B1 at 24 h in the same cells. This was followed by decreased cyclin B1 expression at later time points, paralleled by the appearance of polyploid cells. The prolonged cell cycle arrest in G2/M phase, together with the slower emergence of polyploid cells in the p53-wild type Capan-2, as compared to the p53-mutant AsPC-1, is likely to be related to their distinct p53 status. A similar G2/M cell cycle arrest associated with a transient accumulation of cyclin B1 had been previously described in the p53-wild type A549 lung cancer cell line treated with fenbendazole [26]. In this regard, it has been reported that cell cycle checkpoint protein cyclin B1 accumulates in the presence of aberrant mitosis and that the cyclin is then slowly degraded allowing cell cycle progression [37,38]. In p53-mutant AsPC-1, differently from p53-wild type Capan-2, the quick appearance of polyploid cells after parbendazole treatment was associated to decreased expression of cyclin B1 at all time points. This different behavior might be related to the role of p53 in protecting cells from polyploidization [39]. Taken together, these results suggest that parbendazole markedly alters microtubule organization, induces formation of cells with abnormal spindles and drastically interferes with cell cycle progression by promoting G2/M arrest, followed by the emergence of enlarged, polyploid cells. These abnormalities are referred as mitotic catastrophe by several studies [16,17,37,40]. As previously reported, mitotic catastrophe may culminate in apoptosis [16,17]. In line with this possibility, we tested whether parbendazole could promote apoptosis in PC cells. Consistently, in AsPC-1 and Capan-2 exposed to the drug the increment in the percentage of Annexin-V^pos^ cells showed by flow cytometry and the increased levels of PARP cleavage revealed by immunoblotting indicated that parbendazole induced apoptosis. Remarkably, parbendazole elicited DNA damage response in AsPC-1 and Capan-2 cell lines and also this effect is related to apoptosis induction. According to the role of microtubules in motility [31], parbendazole also hampered AsPC-1 and Capan-2 cell migration. In this regard, considering the important role of microtubules in cells of tumor microenvironment and their implication in survival, drug resistance and invasion in pancreatic and other tumors [34,35,36,41,42], it is possible that parbendazole may affect also the tumor milieu. Notably, several studies indicated that the PC aggressive phenotype could associate to WNT/β-catenin pathway activation, which plays a pivotal role both in invasive and in immunotolerant behavior of PC [43,44,45]. Intriguingly, microtubules are involved in WNT signaling and benzimidazoles have been shown to inhibit WNT/β-catenin signaling pathway [46,47,48]. Thus, it is conceivable that also parbendazole may modulate WNT/β-catenin pathway, with potential effects on immune-homeostasis of PC microenvironment. This might extend the therapeutic window of parbendazole when used alone, or in combination with PC standard chemotherapeutic agents and further studies are needed to address this possibility. Overall, our findings indicate that DNA damage, impairment of cell migration and mitotic catastrophe followed by apoptotic cell death are mechanisms through which parbendazole exerts its anticancer effect in PC cells.

Considering that gemcitabine-based chemotherapy, the mainstay therapy for PC, provides a limited survival advantage for advanced PC patients, we explored the possibility that combinations of parbendazole with gemcitabine might increase the sensitivity of pancreatic cancer cells to this standard chemotherapeutic agent. In this regard, we showed that all the tested combinations induced a dose-dependent decrease of cell viability, with a more enhanced effect at the highest concentrations of the two drugs. It should be noted that most combinations were assessed as synergistic by CompuSyn, supporting the possibility that the combination of parbendazole with gemcitabine might be relevant in the perspective of clinical translation.

An interesting possibility that needs to be considered is that repurposed drugs may act on multiple molecular targets in cancer cells, thus it cannot be excluded that other targets, in addition to tubulin, may emerge for explaining anticancer actions of parbendazole in PC cells. It is also possible that synergistic drug combinations tested in the present study act as new entities exerting antiproliferative effects distinct from those exhibited by individual drugs. Furthermore, drug combinations may affect previously unknown molecular targets, including targets which have been reported to be implicated in human pancreatic cancers, such as epidermal growth factor receptor (EGFR), phosphoinositide 3-kinases/protein kinase alpha/mammalian target of rapamycin (PI3K/Akt/mTOR), cholecystokinin receptors (CCKR), WNT/β-catenin, NOTCH and special AT-rich sequence-binding protein 2 (SATB2) [43,49,50,51,52,53,54], or even novel targets. This possibility will have to be investigated in future studies, in order to explore a potential underlying connection existing among the above-mentioned pathways and their modulation by parbendazole. These studies might expand our insights on the therapeutic relevance of the drug and of its combinations in PC treatment.

## 4. Materials and Methods

### 4.1. Reagents and Antibodies

3-(4,5-Dimethyl-2-thiazolyl)-2,5-diphenyl-2*H*-tetrazolium bromide (MTT), crystal violet, propidium iodide (PI), RNAse, cell lysis buffer, dimethyl sulfoxide (DMSO), phenylmethylsulfonyl fluoride (PMSF), protease and phosphatase inhibitors cocktails were purchased from Sigma-Aldrich (St. Louis, MO, USA). Mouse monoclonal anti-PARP antibody was acquired from Cell Signaling Technology, Inc. (Beverly, MA, USA). Monoclonal anti-β-actin antibody was acquired from Sigma-Aldrich (St. Louis, MO, USA). Anti-phospho-histone H2A.X (Ser139), anti-cyclin B1, goat anti-mouse IgG-horseradish peroxidase (HRP) and goat anti-rabbit IgG-HRP antibodies were purchased from Santa Cruz Biotechnology, Inc. (Dallas, TX, USA).

### 4.2. Cell Lines and Chemicals

Pancreatic cancer (PC) cell lines AsPC-1 and Capan-2 were acquired from Cell Lines Service (CLS, Eppelheim, Germany). AsPC-1 carries mutations both in KRAS and p53, while Capan-2 is KRAS mutated and p53 wild type. Cells were cultured in RPMI 1640 medium, supplemented with 10% fetal bovine serum (FBS) at 37 °C, 5% CO_2_. Parbendazole, fenbendazole, mebendazole and oxibendazole were purchased from Sigma-Aldrich (St. Louis, MO, USA). Gemcitabine was obtained by Selleckchem (Houston, TX, USA). DMSO was used to prepare stock solutions of the different drugs. A fixed amount of DMSO was present in each experiment and the maximum percentage of the solvent in culture media (0.15%) was employed in drug combination experiments.

### 4.3. Cell Viability and Cell Growth Assays

Cell viability was assessed by MTT assay, as previously described [55]. Briefly, cells were seeded in 96-well plates (4 × 10^3^ cells/well) and incubated for 24 h. For initial screening, cells were exposed for 72 h to fenbendazole, mebendazole, oxibendazole or parbendazole at different concentrations as indicated, or to vehicle (DMSO). For combined treatments, experimental design was made according to the Chou-Talalay method for drug combination studies [32]. Specifically, PC cells (4 × 10^3^ cells/well) were treated for 72 h with three concentrations of parbendazole or gemcitabine as single drugs, or with three combinations of the two compounds.

Cell counting was performed using the trypan blue exclusion test. Briefly, PC cells were seeded in 24-well plates (5 × 10^4^ cells/well). After attachment, cells were treated with 0.2 μM, 0.7 μM parbendazole, or with vehicle and counted at 24, 48 and 72 h.

### 4.4. Colony Formation Assay

Colony formation assay was performed as previously described [6]. Briefly, PC cells (1 × 10^3^ cells/well) were seeded in 6-well plates, incubated for 24 h and then treated for 72 h with 0.2 μM, 0.7 μM parbendazole, or with vehicle (DMSO). After 4 d, following methanol fixation and crystal violet staining, colonies of at least 30 cells were counted.

### 4.5. Immunofluorescence

The effects of parbendazole on cell microtubules in AsPC-1 and Capan-2 cells exposed to parbendazole, or to vehicle (DMSO) were examined by confocal microscopy. Briefly, 5 × 10^3^ cells were seeded in 8-well BD Falcon™ CultureSlides (BD Bioscience, San Jose, CA, USA) and incubated overnight at 37 °C, 5% CO_2_, then treated with 0.2 µM, 0.7 µM parbendazole, or with vehicle control (DMSO) for 24 h. Cells were then fixed with 4% paraformaldehyde in phosphate-buffered saline (PBS) for 30 min and washed with PBS. Following fixation, cells were incubated 60 min in 0.3% Triton™ X-100 (Sigma-Aldrich) in PBS+ (PBS, 5% FBS, 0.02% sodium azide and 10 mg/mL BSA), washed with PBS+ and then incubated with the anti-α-tubulin antibody (ab7291, Abcam, Cambridge MA, USA) in PBS+ overnight at room temperature. The primary antibody was detected using the Dylight conjugated anti IgG-heavy and light chain cross-adsorbed species-specific fluorescent secondary antibody (Bethyl Laboratories, Inc., Montgomery, TX, USA). Nuclear staining was performed with DRAQ5 (Cell Signaling Technology, Beverly, MA, USA). Coverslip were mounted with SlowFade^®^ Gold antifade reagent (Thermo Fisher Scientific, Life Technologies Italia Fil., Monza MB, Italia). All the images were acquired with identical settings and corrected by background subtraction using a Carl Zeiss LSM5 Pascal confocal laser scanning microscope (Carl Zeiss 40X Plan Neofluar oil-immersion objective, Carl Zeiss Microscopy, LLC, NY, USA).

### 4.6. Cell Cycle Analysis

To perform cell cycle analysis, PC cells (0.3 × 10^6^) were collected, fixed in 70% cold ethanol and kept at 4 °C overnight. Then, cells were suspended in 5 µg/mL PI (final concentration) and 100 µg/mL RNAse (final concentration), and incubated at 4 °C overnight. Cell cycle profiles were analyzed as previously described [55].

### 4.7. Apoptosis Assay

Apoptosis assay was performed essentially as previously described [55]. Analyses were performed using a CytoFLEX flow cytometer with the FL1 detector (linear mode) and the CytoExpert software (Beckmann Coulter, Milano, Italy). More than 10^4^ events were collected for each sample.

### 4.8. Western Blotting Analysis

PC cell lines treated with 0.2 μM, 0.7 μM parbendazole, or with vehicle (DMSO) were lysed in a cell lysis buffer containing PMSF, protease inhibitors cocktails and phosphatase inhibitors. Protein lysate quantification and immunoblot analyses were performed as previously described [55].

### 4.9. Wound-Healing Assay

PC cells were seeded in 12-well plates (1 × 10^6^ cells/well) and incubated in medium containing 10% FBS to allow the formation of a complete monolayer. Scratches were generated at the center of each well using sterile pipette tips. To remove the detached cells, plates were washed using PBS, and subsequently, they were incubated with serum-free medium containing 0.2 μM, 0.7 μM parbendazole, or vehicle. To analyze the dynamics of wound closures, pictures were taken immediately after scratching (0 h) and at the indicated time intervals (6 and 24 h post-scratching), until the wound closure was completed by cells treated with vehicle. Relative scratch gaps were calculated as the ratio between the remaining gap at a given time point and the corresponding gap at 0 h.

### 4.10. Calculation of IC_50_ and Combination Index (CI)

IC_50_ values were extrapolated from dose-response curves and calculated using the CompuSyn software. The interactions between parbendazole and gemcitabine were assessed by calculating the CI values using the CompuSyn software [32]. Based on this analysis, a CI < 1 indicates synergism, a CI = 1 indicates additive effects and a CI > 1 indicates antagonism.

### 4.11. Statistical Analysis

Data were expressed as the mean ± standard deviation. For cell viability, the distribution of samples was normal and statistical analysis was performed by one-way ANOVA followed by Dunnett’s test for multiple comparisons. For other analyses, considering the sample size the independent samples t-test was used to compare treated to control samples [56].

## 5. Conclusions

This is the first study showing that the FDA-approved parbendazole has a prominent antitumor activity in PC cells. Parbendazole markedly inhibited viability, proliferation, clonogenicity and cell migration of PC cell lines through mechanisms involving DNA damage, alteration of microtubule organization, formation of irregular mitotic spindles and profound cell cycle perturbation. These effects were consistent with the promotion of mitotic catastrophe in PC cell lines, which culminated in apoptosis. Notably, parbendazole in combination with gemcitabine synergistically affected PC cell viability, providing a rationale for its potential repurposing in the currently dismal treatment of pancreatic cancer.

## Supplementary Materials

The following are available online at https://www.mdpi.com/2072-6694/11/12/2042/s1, Figure S1: Full-length blots of Figure 4, panel D, Figure S2: Full-length blots of Figure 5, panel B, Figure S3: Full-length blots of Figure 5, panel C.
