# Supplementary material for: The Benzimidazole-Based Anthelmintic Parbendazole: A Repurposed Drug Candidate That Synergizes with Gemcitabine in Pancreatic Cancer
Source: Cancers (Basel). 2019 Dec 17;11(12):2042. doi: 10.3390/cancers11122042 (PMC6966614; doi:10.3390/cancers11122042)
Supplement: Supplementary file 1 [file cancers-11-02042-s001.pdf]

Supplementary Materials: The Benzimidazole-Based Anthelmintic Parbendazole: A Repurposed Drug Candidate That Synergizes with Gemcitabine in Pancreatic Cancer

Rosalba Florio, Serena Veschi, Viviana di Giacomo, Sara Pagotto, Simone Carradori, Fabio Verginelli, Roberto Cirilli, Adriano Casulli, Antonino Grassadonia, Nicola Tinari, Amelia Cataldi, Rosa Amoroso, Alessandro Cama and Laura De Lellis

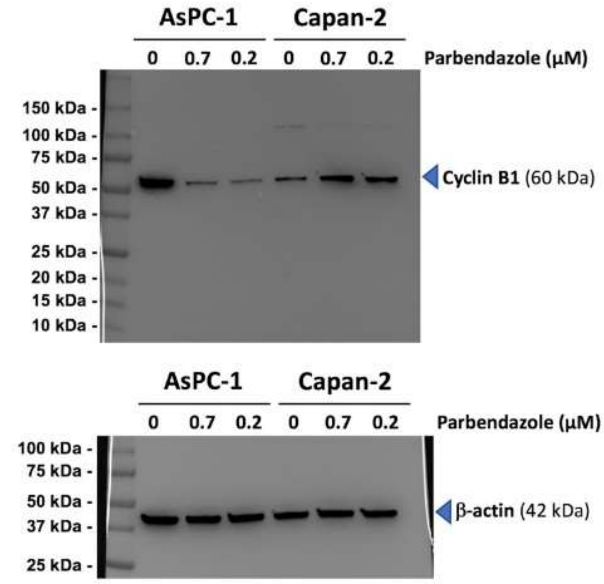

A

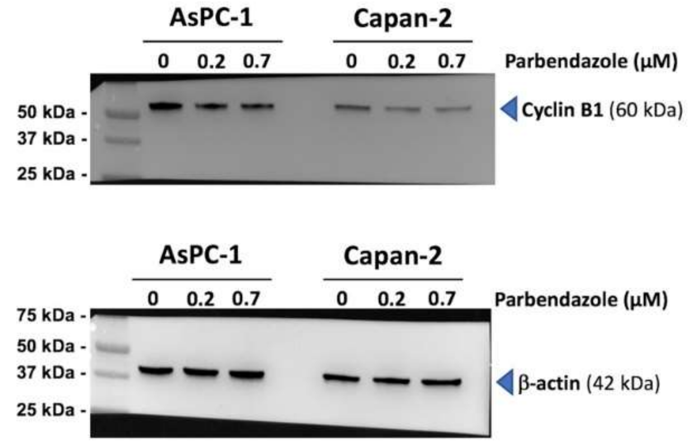

B

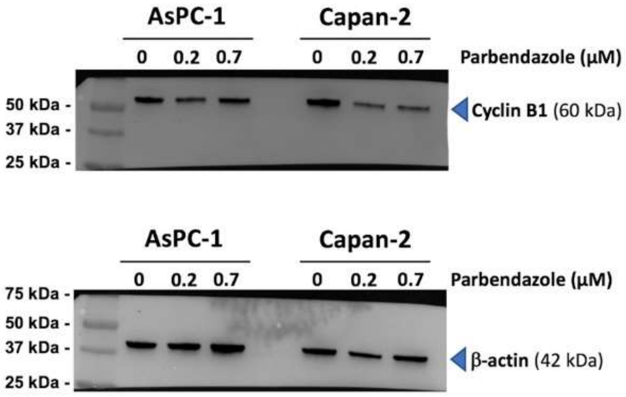

C

Figure S1. Full-length western blots of cyclin B1 and β-actin in AsPC-1 and Capan-2 pancreatic cancer cell lines, treated with 0 µM, 0.2 µM or 0.7 µM parbendazole for 24 h (panel A), 48 h (panel B) and 72 h (panel C) (the

corresponding cropped blots are shown in Figure 4D of the main text). Each full-length membrane was incubated with the first antibody, then stripped and reprobed with the next indicated antibody.

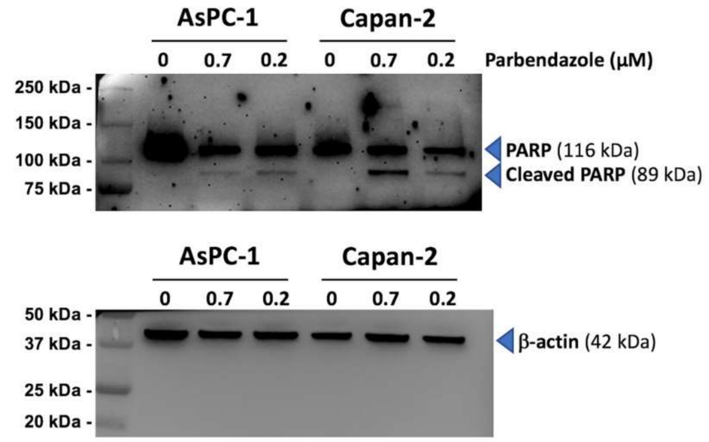

Figure S2. Full-length western blots of PARP, cleaved PARP and β-actin in AsPC-1 and Capan-2 pancreatic cancer cell lines, treated with 0 µM, 0.2 µM or 0.7 µM parbendazole (the corresponding cropped blots are shown in Figure 5B of the main text). The full-length membranes were cut and incubated with the indicated antibodies.

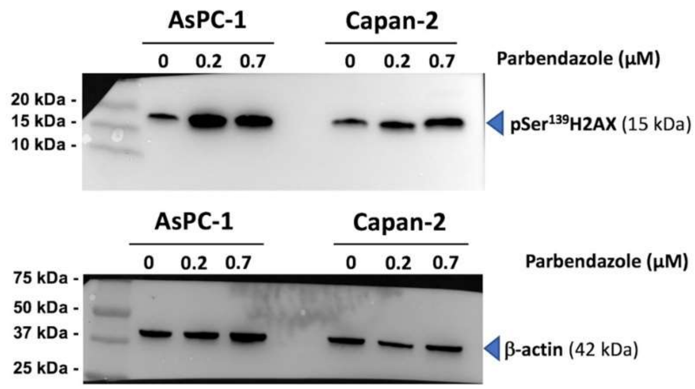

Figure S3. Full-length western blots of pSer^{139}H2AX and β-actin in AsPC-1 and Capan-2 pancreatic cancer cell lines, treated with 0 µM, 0.2 µM or 0.7 µM parbendazole (the corresponding cropped blots are shown in Figure 5C of the main text). The full-length membranes were cut and incubated with the indicated antibodies.

© 2019 by the authors. Licensee MDPI, Basel, Switzerland. This article is an open access article distributed under the terms and conditions of the Creative Commons Attribution (CC BY) license (http://creativecommons.org/licenses/by/4.0/).
